# Effectiveness of a tailored, integrative Internet intervention (deprexis) for depression: Updated meta-analysis

**DOI:** 10.1371/journal.pone.0228100

**Published:** 2020-01-30

**Authors:** Conal Twomey, Gary O’Reilly, Oliver Bültmann, Björn Meyer

**Affiliations:** 1 School of Psychology, University College Dublin, Belfield, Dublin, Ireland; 2 Research Department, Gaia, Hamburg, Germany; 3 Department of Psychology, City, University of London, London, England, United Kingdom; Simon Fraser University, UNITED STATES

## Abstract

Digitally delivered interventions for depression vary in many aspects, including their therapeutic orientation, depth of content, interactivity, individual tailoring, inclusion of multimedia, cost, and effectiveness. However, their effectiveness is rarely examined in intervention-specific meta-analyses. An earlier meta-analysis of eight randomized controlled trials (RCT) demonstrated the effectiveness of a tailored, integrative digital intervention (deprexis), which is delivered via the Internet. This updated meta-analysis of twelve deprexis-specific RCT with a total of *N* = 2901 participants confirmed the effectiveness of deprexis for depression reduction at post-intervention (*g* = 0.51, 95% CI: 0.40–0.62, *I*^2^ = 26%). Results were analogous when study quality, screening and randomization procedure were taken into account. Clinician guidance, developer-involvement, setting (community vs. clinical), and initial symptom severity did not have statistically significant effects on the effect size, and there was no evidence of publication bias. Thus, these findings demonstrate that deprexis can facilitate clinically relevant reduction of depressive symptoms over 8–12 weeks across a broad range of initial symptom severity, and that the intervention can be combined with other forms of depression treatment. There is now a need to study the intervention’s implementation in routine care settings as well as its long-term effectiveness and cost-effectiveness in diverse cultural and linguistic settings.

## Introduction

The global treatment gap for depression ranges from 72% in high-income countries to 93% in low-income countries, and initiatives to scale up effective treatments are likely to be cost-effective because untreated depression is often associated with poor health and increased disability risk [1]. Internet-based depression treatments are promising in this regard because they can be scaled up easily, and recent meta-analyses have shown that even self-guided Internet interventions can achieve clinically relevant effects on depressive symptom reduction (g = 0.27, NNT = 6.58) [2], which may be augmented when additional clinician support is provided [3].

Despite such encouraging findings, caution is warranted for several reasons, and sweeping conclusions suggesting that all Internet interventions for depression are effective would be misguided. Of note, meta-analytic evidence showing that such interventions are effective on average does not mean that these interventions are all equally effective or, indeed, that any one intervention is effective at all. Internet interventions for depression differ substantially in their accessibility, therapeutic orientation, depth of content, interactivity, individual tailoring, inclusion of multimedia, self-monitoring tools, homework assignments, and cost [4]. Worryingly, many such interventions for depression are not evidence-based because they are never evaluated in randomized clinical trials (RCTs); this was the case for 20 out of 32 identified programs in a recent review [4].

Given this substantial heterogeneity among Internet interventions for depression, one might expect that they also differ in terms of their effectiveness, although head-to-head trials are rare. In one large trial that included two Internet interventions for depression (Beating the Blues and MoodGYM), neither of them was effective when added to primary care in the United Kingdom [5]. A recent meta-analysis of self-guided depression-focussed Internet interventions yielded a modest average effect of Hedges’ *g* = 0.27 across all programmes, but the effects of individual interventions ranged from -0.13 to 0.89, and there was significant moderate to high heterogeneity, suggesting that these interventions were not equally effective.

To examine the effectiveness of specific Internet interventions for depression, two intervention-specific meta-analyses have recently been completed. The first of these reported an average effect of *g* = 0.36 for MoodGYM, an Australian intervention based on cognitive behaviour therapy (CBT). Even though this average effect is above the recently proposed threshold of clinical relevance of *d* = 0.24 [6], the effect shrank to a non-significant level of *g* = 0.17 after adjusting for publication bias. Furthermore, moderator analyses suggested that the intervention was more effective in trials set in Australia compared to Europe-based trials, and larger effects were found when face-to-face guidance was provided, compared to remote guidance (telephone or email). Of note, 11 of the 12 included trials examined the intervention with some form of personal guidance, making it impossible to disentangle whether the intervention effects could be attributed to guidance or to the online programme, as such [7].

A second intervention-specific meta-analysis recently reported an average effect of *g* = 0.54 for deprexis, an integrative depression-focussed Internet intervention developed in Germany. This intervention differs in many ways from MoodGYM but is also broadly based on CBT principles. Unlike MoodGYM, deprexis also contains interactive modules on topics such as positive psychology, interpersonal skills, or coping with difficult childhood memories; more detailed descriptions are provided in several other articles [8–11]. The deprexis intervention is also unique in the sense that it engages users in ‘simulated dialogues’, such that users can continuously select from predefined response options, and then custom-tailors subsequent content to individual user needs and preferences. The deprexis-specific meta-analysis was based on eight RCTs that had been published up to November of 2016.

The purpose of the present meta-analysis is to provide an update of the deprexis-specific meta-analysis, which seems important because four more trials have been published since then, including two that are unique because they examined the utility of adding deprexis adjunctively to inpatient or outpatient depression treatment. Furthermore, one of these new trials used an active control condition (a psychoeducational depression-focussed online programme), which is still rare because most trials of behavioural interventions for psychiatric conditions continue to use waitlist or treatment-as-usual control conditions [12, 13]. As in the previous meta-analysis, we examined several potential moderators of treatment effects, including the setting (clinical, community), developer-involvement, and provision of personal support. Additionally, we examined potential publication bias.

## Methods

### Eligibility criteria, literature search, data extraction and quality ratings

The present study was modelled closely after the previous deprexis-specific meta-analysis and, therefore, used the same eligibility criteria for study selection and equivalent literature search, data extraction and quality assessment methods [11]. In brief, all studies were eligible in which deprexis was used as an intervention for adults with elevated depression symptoms, and which included any kind of control condition. Waiting lists, delayed programme access, no treatment, treatment as usual (TAU), or active control interventions (computerized or not) were eligible. Only randomized controlled trials (RCTs) that focussed on the outcome of self-reported or clinician-rated depression measures were included, and any language was permitted.

### Literature search and data extraction

We used the same search term (“deprexis”) that was used in the previous deprexis-specific meta-analysis [11] and searched five databases: PsycINFO, CINAHL Complete, MEDLINE, Science Direct and Web of Science. The final search was conducted on August 14^th^, 2019. We hand-searched reference lists of included articles to identify additional articles. Screening of all articles were undertaken by two authors (BM and OB), and data were managed using EndNote X7 (Thomson Reuters Corp.) and word processing software. For each study, we recorded the setting, country, screening method (e.g., questionnaire cut-off or diagnostic interview), demographic data, control condition, randomization method, guidance (none, clinician, technician), outcome measures, assessment time-points, trial dropout at primary endpoint, and treatment outcomes.

### Quality assessment

As in the previous deprexis-specific meta-analysis [11], we used three of the seven quality criteria stipulated by the Cochrane Collaboration’s tool for the assessment of risk of bias [14]: random sequence generation, allocation concealment, and completeness of outcome data (when intention-to-treat analyses were used, data were deemed complete). Blinding from intervention knowledge was not used because the experimental conditions did not permit such blinding; blinding of outcome assessment was also not used because only self-report assessments were included; and selective reporting bias as well as other bias were deemed too ambiguous and, as in the previous meta-analysis, were not included as quality criteria. Only information described in the published articles was used in the quality ratings. Of note, the lead author of one of the studies that was included in both the previous deprexis meta-analysis and the current meta-analysis has questioned the quality ratings assigned to that study [15]; however, our response to these concerns has been published [16], and we decided to not alter the original quality ratings for the eight studies included in both meta-analyses.

### Statistical analysis

Meta-analysis using a random effects model was performed, and all statistical analyses were conducted with *Comprehensive Meta-Analysis* (version 3.3.070). We calculated pooled mean effect sizes (Hedges’ *g*) with 95% confidence intervals (CI); following conventions, we regarded effect sizes of 0.2 as small, 0.5 as moderate, and 0.8 as large. Higgin’s *I*^2^ percentages were calculated to estimate heterogeneity and values of 25%, 50% and 75% were regarded as low, moderate and high heterogeneity, respectively. We analysed only data at the post-intervention time-point (8–12 weeks) as this was the only assessment time-point in most trials, and control participants often received access to the intervention after this time-point. As in the previous meta-analysis, when there was more than one study arm in which deprexis was used, the effects of these multiple arms were averaged and entered only once in the analysis to avoid double-counting.

We also performed sensitivity analyses in which we adjusted for study quality, depression screening, randomization method and publication bias. The ‘Trim and Fill’ procedure [17] was used to examine potential publication bias. Subgroup analyses were conducted to examine (a) whether the provision of personal guidance, (b) developer-involvement, (c) the setting from which the sample was recruited (community versus clinical treatment), (d) publication date, or (e) initial depressive symptom severity influenced the results.

## Results

### Study selection

**Fig 1:**
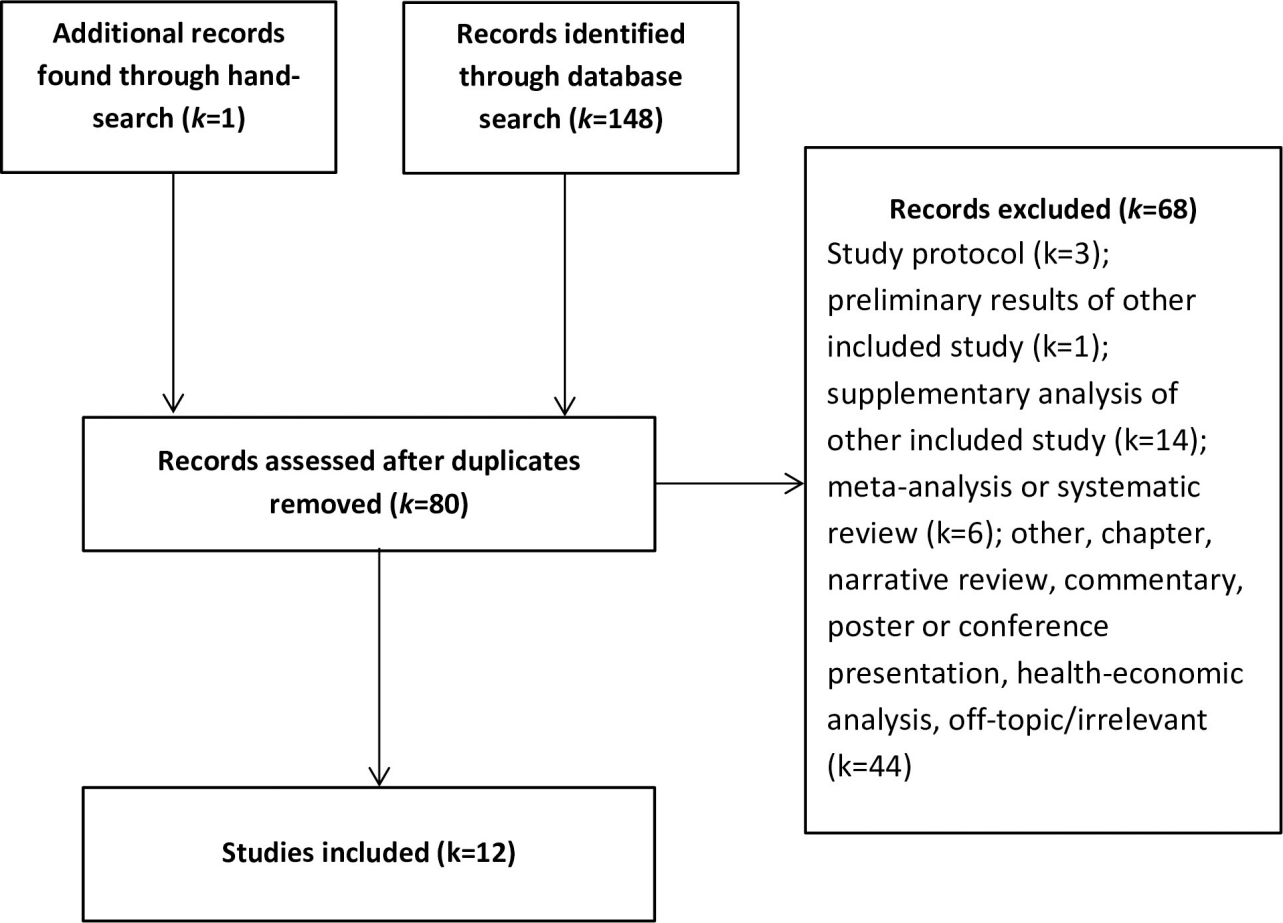
Literature search flow.

Fig 1 shows the literature search flow. A total of 80 abstracts were screened after removal of duplicates. Screening agreement rate between the authors BM and OB was 100%. After excluding records because they were not RCTs, 12 studies were included in the meta-analysis [8–10, 18–26].

### Study characteristics

Table 1 summarizes the study characteristics. In 5 of the 12 studies (42%), a deprexis developer was involved in the study and listed as an author. Whereas 10 studies recruited participants primarily from the community with invitational advertisements, 2 studies recruited patients from clinical treatment settings, including outpatient psychotherapy and multimodal inpatient depression treatment at a psychosomatic hospital. Seven of the 12 studies formally screened for the presence of elevated depressive symptoms or a diagnosis of a depressive disorder, whereas 5 studies required only subjective feelings of sadness or depression or did not screen participants for depressive symptoms. Sample sizes ranged from 27 to 1013, resulting in a total of 2901 across all studies. Except for one study [20], all other studies included more women than men, and the mean age ranged from 32 to 48.

**Table 1:** Study characteristics.

Study	Setting (country)	Depression screening	Baseline depression	N	%f	M Age (SD)	Control	Randomization ratio	Guidance	Outcome		Dropout (%)		Quality		
			severity								PI time (weeks)	DEPR	Ctr	R	A	C
Beevers (2017)*	Community (USA)	Moderate depression	Moderate	376	74	31.9 (11.2)	WL	3:1 (in favour of DEPR)	Emails and phone calls	QIDS	8	22	11	+	+	+
Berger (2011)^2^	Community (Switzerland, Germany)	MDD or dysthymia	Severe	76	53	38.8 (14.0)	WL	1:1	None or emails	BDI-II	10	6	15	+	+	+
Fischer (2015)	Clinical and community (MS, Germany)	Indication of depression	Mild	90	78	45.3 (11.6)	WL	1:1	None	BDI-II	9	22	20	+	+	+
Klein (2016)*	Clinical and community (Germany)	Mild-to-moderate depression	Moderate	1013	69	42.9 (11.0)	WL	1:1	Emails	PHQ-9	12	22	21	+	+	+
Meyer (2009)*	Clinical and community (Germany)	None	Moderate	396	76	34.8 (11.6)	WL	4:1 (in favour of DEPR)	None	BDI-I	9	50	54	+	-	+
Meyer (2015)*	Clinical and community (Germany)	Severe depression	Severe	163	75	42.0 (11.39)	WL	1:1	None	PHQ-9	9	22	14	+	+	+
Moritz (2012)	Community (Germany)	None	Moderate	210	79	38.5 (13.29)	WL	1:1	None	BDI-I	8	22	14	-	-	+
Schröder (2014)	Clinical and community (epilepsy, Germany)	None	Moderate	78	75	37.5 (10.92)	WL	1:1	None	BDI-II	9	34	20	+	+	+
Berger (2018)*	Clinical (psychotherapy, Germany)	Moderate depression	Severe	98	66	43.05 (12.10)	Psychoth.	1:1	Psycho-therapists	BDI-II	12	27	32	+	+	+
Bücker (2018)	Community (slot machine gamblers, Germany)	Feelings of sadness and desperation	Moderate	145	24	35.73 (10.14)	WL	1:1	None	PHQ-9	8	56	39	+	+	+
Fuhr (2018)	Community (psychotherapy waitlist, Germany)	Depressive symptoms	Moderate	27	67	37.83 (13.27)	PSY-WL	1:1	None	PHQ-9	10	15	7	+	-	-
Zwerenz (2017)	Clinical (inpatient hospital, Germany)	Moderate depression	Severe	229	61	47.98 (9.79)	Active (online info)	1:1	Inpatient staff	BDI-II	12	26	25	+	+	+

*deprexis developer is an author. Dropout (%) from study at post-intervention.

^2^Results from two deprexis treatment arms (with, and without, clinician or technician guidance) were averaged together for the current analysis. %f = % female in sample; Ctr = Control; DEPR = deprexis; f = % female; M = Mean; MS = Multiple sclerosis; PI = post-intervention data collection point; SD = standard deviation. Measures: BDI-II = Beck Depression Inventory-II; BDI-II = Beck Depression Inventory-II; HAQUAMS = Hamburg Quality of Life Questionnaire for Multiple Sclerosis; PHQ-9 = Patient Health Questionnaire- 9; QIDS = Quick Inventory of Depression Symptoms. Quality: A = allocation concealment; C = completeness of data; R = random sequence generation.± = Procedure to minimise bias reported/ not reported; WL = on waiting list to access deprexis but access to other care-as-usual (TAU) options; PSY-WL = on waiting list (to start outpatient psychotherapy) and also access to TAU. ^§^ The lead author has disagreed with our quality rating and has argued that positive ratings should be assigned on all three quality criteria [15]; our response to this disagreement has been published [16], and we decided not to alter these quality ratings.

None of the studies prohibited access to other care options, meaning that deprexis was always used adjunctively to treatment as usual (TAU). Control conditions were, therefore, usually a combination of heterogeneous TAU and waiting list in the sense that control group participants typically received access to deprexis after the post-intervention time-point. In one study [26], an active control condition was used (12 sessions of online psychoeducational material). In two studies [19, 26], all participants received structured depression treatments (outpatient psychotherapy, multimodal inpatient psychotherapy), and deprexis was used adjunctively to these treatments in the intervention groups only.

Self-report instruments of depressive symptom severity served as primary outcome measures in all studies, although some studies also included clinician-rating measures as secondary outcomes. Post-intervention data collection time-points ranged from 8 to 12 weeks, and study dropout rates in the interventions conditions ranged from 6% to 56%, with a weighted average of 27.84%. Eight of the 12 studies met all three quality criteria. We did not change the quality ratings reported for the eight studies in the earlier deprexis-specific meta-analysis [11].

Of the four new studies, the one study with lower quality ratings was somewhat of an outlier in several aspects: (a) it was published in German, (b) it was described by the authors as a pilot study rather than a full-scale RCT, (c) it was not registered in a trial registry, (d) it did not report intention to treat (ITT) analyses but only analyses based on completer data, (e) it was statistically underpowered because only 14 and 13 participants were in the intervention and control groups, respectively, which corresponds to statistical power of 0.24 for the detection of an effect of *d* = 0.5, based on a post-hoc power-analysis performed with G*Power 3.1 [27]). The descriptions of the randomization process and the concealment of allocation were not detailed enough to permit positive ratings on these dimensions, in our judgment.

### Effectiveness of deprexis for reducing depressive symptoms

**Fig 2:**
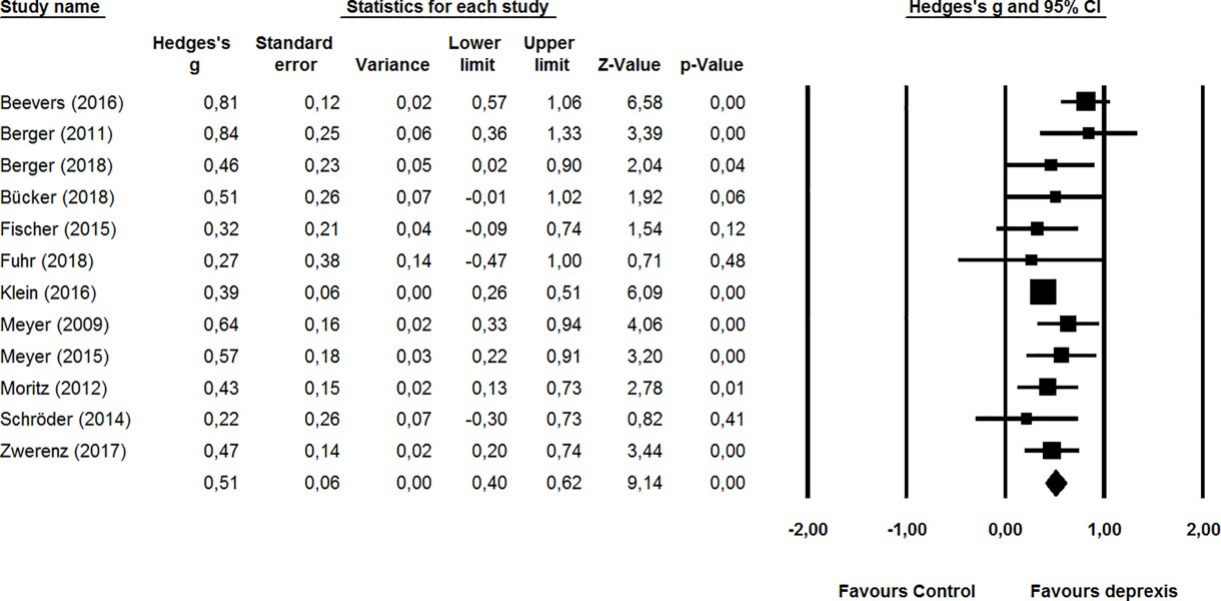
Forest plot.

As shown in the forest plot (Fig 2), comparisons from 12 studies demonstrated the effectiveness of deprexis for depressive symptoms at post-intervention, with an effect size of *g* = 0.51 (95% CI: 0.40–0.62) and low heterogeneity (*I*^2^ = 26%).

**Fig 3:**
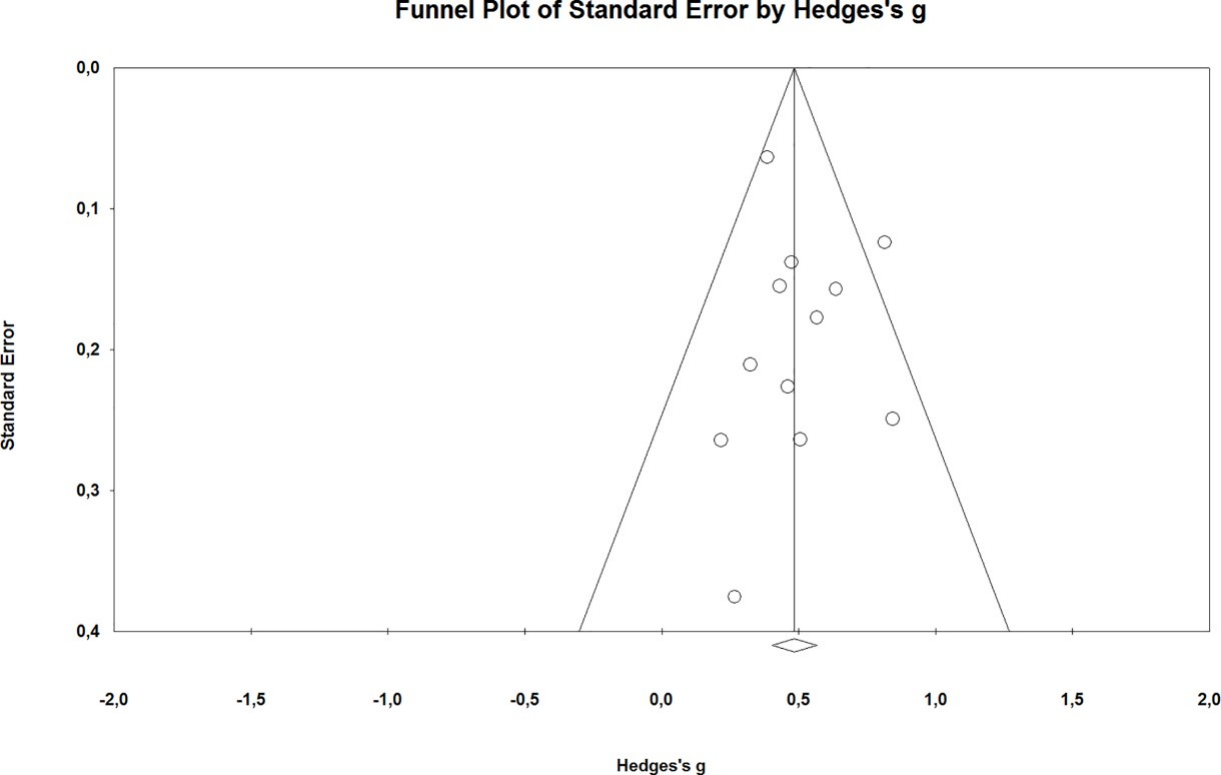
Funnel plot.

Sensitivity analyses showed that removing lower quality studies (*k* = 3; Fuhr et al., 2018; Meyer et al., 2009; Moritz et al., 2012) did not reduce the effect size (*g* = 0.52, 95% CI: 0.38–0.66; *I*^2^ = 41%). Removing the two studies with weighted randomization did reduce the effect size slightly but not significantly (*g* = 0.42, 95% CI: 0.32–0.51; *I*^2^ = 0%). The funnel plot was symmetrical, indicating no publication bias (Fig 3).

Subgroup analyses showed that effect sizes were slightly larger when deprexis was provided with some form of clinician guidance, but the difference between guided versus unguided intervention use was not statistically significant. The effect size in studies with guidance (*k* = 5) was *g* = 0.57, 95% CI: 0.36–0.78; *I*^2^ = 66%, whereas the effect in studies without guidance (*k* = 7) was *g* = 0.47, 95% CI: 0.32–0.62; *I*^2^ = 0%. The effect size did not differ significantly between studies that involved intervention developers (*k* = 5) compared to those that did not (*k* = 7). The effect size in studies with developer-involvement was *g* = 0.57, 95% CI: 0.37–0.76; *I*^2^ = 62%, whereas in studies without developer-involvement it was *g* = 0.45, 95% CI: 0.30–0.60; *I*^2^ = 0%. Of note, 3 of 5 studies (60%) with developer-involvement also included clinician guidance, whereas this was true of only 2 of the 7 studies (29%) without developer-involvement. Thus, the slightly but non-significantly larger effects in studies with developer-involvement could be attributed to the provision of clinician support, which was also associated with slightly but non-significantly larger effects.

The effect size in the two studies that recruited patients from clinical settings (outpatient psychotherapy or inpatient depression treatment) did not differ significantly from studies that recruited participants from the community. The effect size in studies with participants recruited from the community was *g* = 0.52, 95% CI: 0.39–0.66; *I*^2^ = 39%, whereas it was *g* = 0.47, 95% CI: 0.24–0.70; *I*^2^ = 0% in studies with participants from clinical settings. The effect sizes observed in the four newly included studies was *g* = 0.46, 95% CI: 0.26–0.67; *I*^2^ = 0%, which did not differ significantly from the 8 studies already included in the previous meta-analysis, *g* = 0.53, 95% CI: 0.38–0.69; *I*^2^ = 52%. The effect size in eight studies that included samples with mild (*k* = 1) or moderate baseline depression severity (*k* = 7) was *g* = 0.49, 95% CI: 0.34–0.65; *I*^2^ = 43%, whereas in four studies with severely depressed samples, it was *g* = 0.55, 95% CI: 0.37–0.73; *I*^2^ = 0%. These effects did not differ significantly from each other.

## Discussion

### Summary of main findings

This meta-analysis of twelve studies confirmed the effectiveness of deprexis for the reduction of depressive symptoms over a period of 8 to 12 weeks, with an effect size of *g* = 0.51 (95% CI: 0.40–0.62). This effect size is well above the threshold for clinical significance, which has been proposed at the level of *d* = 0.24 [6]. The effect of *g* = 0.51 can be converted to a number-needed-to-treat (NNT) of 3.55, which may be a metric more familiar to many psychiatrists than Hedges’ *g* or Cohen’s *d* [28]. Thus, between 3 and 4 individuals would have to be treated with deprexis in order to achieve clinically relevant depression reduction that would not have occurred otherwise.

The effect size estimate is robust in the sense that sensitivity analyses showed that removing lower quality studies or studies with weighted randomization did not significantly alter this effect. Additionally, there was no publication bias as the funnel plot was symmetrical, and there was no developer-bias, as studies that were conducted independently did not differ significantly from studies in which developers were involved.

In line with the earlier deprexis meta-analysis, this updated meta-analyses showed a slight but not statistically significant difference between studies with versus without clinician guidance (*d* = 0.57 vs. 0.47). Thus, this body of evidence indicates that deprexis is associated with clinically relevant effects on depression reduction, even when no clinician guidance is provided. Further research is required to clarify under which conditions the provision of personal support could augment the intervention’s effectiveness.

In contrast to the previous meta-analysis, the present study also showed clinically relevant effects when deprexis was used as an adjunctive treatment to outpatient psychotherapy or multimodal inpatient depression treatment. Indeed, the effect size observed in these studies conducted in clinical treatment settings did not differ from that observed in studies with participants from community settings. In one of these studies, control group patients not only received inpatient treatment, which included face-to-face psychotherapy, but also an active control programme that included 12 sessions of depression-related psychoeducational content [26]. In that study, statistically significant and clinically relevant intervention effects on depression reduction were shown at discharge from the hospital and at 3 and 6 months follow-up [26, 29]. Consistent with meta-analytic evidence [30], deprexis was found to be equally effective in samples with mild to moderate and even severe depressive symptom severity. Thus, the intervention is relevant for patients with a broad range of symptom severity, and it can be combined with other treatments, including inpatient and outpatient psychotherapy as well as antidepressant medication. In severe depression, the combination of deprexis and antidepressants may yield particularly large effects [9].

### Limitations and strengths

With 12 studies and a total of 2901 participants, this is the largest meta-analysis to date of the Internet intervention deprexis, and one of the largest of any depression-focused Internet intervention. Of note, even though the studies were conducted with a wide range of samples in various settings, heterogeneity was low (*I*^2^ = 26%), suggesting that this intervention has a relatively uniform effect across settings and populations. Whereas previous findings had been limited to adults recruited from community settings, the present analyses also included two methodologically sound studies with samples recruited from clinical treatment settings. The evidence showed that using deprexis adjunctively to outpatient therapy or inpatient depression treatment is effective, with effect sizes resembling those observed in community-based studies. A finding that is particularly relevant to clinicians with limited time is that deprexis appears to be effective even without the provision of clinician support; further research is needed to confirm this tentative yet promising finding. A limitation of the present analyses is that the focus was limited to short-term outcome, as we only analysed data collected between 8 and 12 weeks after baseline. Several studies have shown longer term effects of deprexis [8, 9, 23, 29], but for the purposes of this meta-analysis there was still an insufficient amount of data to examine the stability of intervention effects over six months or beyond.

The deprexis intervention is currently available in nine languages, but 11 of the 12 studies included in this meta-analysis were conducted in German-speaking countries and only one was set in the United States [10]. Of note, the post-treatment effect in that study was the second largest of all included studies (*g* = 0.81), even though only minimal personal support was provided (on average, one brief phone call and one e-mail over a period of eight weeks, only to participants who failed to engage with deprexis or complete questionnaires across a two-week period). Thus, further research is needed to replicate these promising results and examine the effectiveness of deprexis in different linguistic and cultural settings.

### Comparisons with other studies

The effect size of *g* = 0.51 (NNT = 3.55) we observed in this meta-analysis is in line with a previous deprexis-specific meta-analysis that included only 2/3 of the 12 studies we examined. The data showed that the new studies did not yield different effect sizes than earlier studies, on average, suggesting that the intervention effect has not declined in recent years. By contrast, there is some evidence suggesting that the effects of certain psychotropic drugs or face-to-face CBT may be declining in more recent studies [31–33].

The current results also compare favourably in the context of other depression-focussed Internet interventions. For example, a meta-analysis on the effectiveness of MoodGYM yielded a smaller effect size of *g* = 0.36 (NNT = 5), which was reduced to a non-significant *g* = 0.17 (NNT = 10.4) after correcting for publication bias [7]. Additionally, this meta-analysis found that larger effects were only observed for MoodGYM in studies that involved face-to-face guidance, whereas effects were small (*g* = 0.23, NNT = 7.7) when remote guidance by telephone or email was provided, and there was no evidence to suggest that the programme was effective without any guidance. However, guidance does not always enhance the effects of Internet interventions, as several trials have not found larger effects when guidance was provided, and a meta-analysis suggested that the effects of guidance may be less pronounced than often assumed [34–36]. In our meta-analysis, the effect sizes for deprexis were in the clinically relevant range regardless of whether no guidance or some guidance was provided.

There is also broader evidence to suggest that an effect size of *g* = 0.51 (NNT = 3.55) compares favourably to other depression interventions, whether delivered via the Internet or not. For example, an individual patient-level meta-analysis of self-guided Internet interventions for depression yielded an average effect of *g* = 0.27 (NNT = 6.58) [2]. However, there was considerable heterogeneity (*I*^2^ = 71%), and study-specific effect sizes varied substantially. Indeed, 5 of the 13 studies in the meta-analysis by Karyotaki and colleagues had examined deprexis, and their average effect size was *g* = 0.58 (NNT = 3.14), contrasting with *g* = 0.28 (NNT = 6.41) for the other interventions. An effect of *g* = 0.51 compares favourably even to treatment with antidepressant medication or different types of psychotherapy, especially after correcting for publication bias and other methodological biases, such as investigator allegiance and type of control group, which may artificially inflate effects [6, 37–39]. For example, after adjusting for methodological biases, the effect of psychotherapy for depression has recently been estimated at *g* = 0.31 (NNT = 5.75) The same effect size of *g* = 0.31 has been reported for antidepressant medication after adjusting for publication bias [40], and the effect of combined psychotherapy and pharmacotherapy for depression has been estimated at *g* = 0.46 (NNT = 3.91) [6].

When comparing these effect sizes, it is important to keep in mind that control conditions often differ in trials investigating medication, psychotherapy, or Internet interventions, and these differences in comparators may influence the magnitude of effect sizes. Specifically, whereas antidepressants are often compared to pill placebo conditions, psychotherapy and Internet interventions are often compared to treatment as usual (TAU) or wait-list conditions [41–43]. In part, this may be due to the problem that constructing a “psychotherapy placebo” is fraught with conceptual difficulties, such as developing a seemingly plausible psychotherapeutic intervention that actually contains no “active ingredients” of psychotherapeutic effectiveness [44, 45]. Nevertheless, it is possible to compare psychotherapy or Internet interventions to more active control conditions than TAU or wait-list, and two deprexis studies have done so. In both of these studies, the effect of adjunctive use of deprexis was compared with relatively intensive forms of depression treatment (outpatient or inpatient depression treatment), and both trials yielded clinically relevant effects in favour of deprexis [19, 26]. Moreover, one of these studies [26], which was developer-independent, used an active control intervention (12 psychoeducational modules on depression), suggesting that the intervention effects are not limited to comparisons with TAU or wait-list control conditions.

The average dropout rate of 27.8% observed in this meta-analysis was lower than that reported in the MoodGYM meta-analysis (41.3%). Conceivably, dropout rates were relatively low because deprexis, as a tailored programme, adapts its content to individual users, which might provide a personalized experience that motivates them to continue. Of note, though, dropout rates varied drastically between different studies. In one study that involved weekly email contacts with clinicians, the dropout rate was zero as every participant completed post-intervention assessments, whereas in another study of mostly male slot machine gamblers with comorbid depression, a dropout rate as high as 56% was reported. A previous individual patient meta-analysis that included data from several deprexis studies suggested that male gender, lower educational level and comorbid anxiety predicted dropout risk [46], whereas a large deprexis trial showed that older age and greater depressive symptom severity were associated with reduced dropout risk [47]. Despite these intriguing emerging findings, more research is needed to illuminate the factors that predict adherence and dropout risk. Clinicians wishing to use deprexis in their practice would be well advised to consider these findings, anticipate adherence barriers, and take measures to minimize dropout risk.

### Implications for clinical practice and research

In the previous deprexis meta-analysis, a call to action was to conduct more research on deprexis in patient samples drawn from clinical settings rather than general community samples. As noted above, this has now been addressed in two studies, and one of these recently reported that the clinically relevant effects observed at three months were still noted and even larger at 6 month follow-up [29]. Nevertheless, more research on the programme’s effectiveness in routine clinical settings, on the long-term stability of effects, health-economic effects, and on the applicability in different populations and global regions is still needed. Emerging evidence suggests that deprexis can be cost effective in the sense that its use may reduce the need for other costly treatments over time [48, 49]. Additionally, more is becoming known about treatment moderators, mediators, response predictors and patterns of change [50–54]. With this increasingly differentiated body of evidence, it should become possible to deploy interventions such as deprexis in such a way that clinical benefits for individual patients are maximized while minimizing costs and staff burden.

Given the fact that the vast majority of people affected by depression still do not have access to effective treatments, these findings suggest that effective and scalable interventions, such as deprexis, should be disseminated more broadly in order to help mitigate the treatment gap and improve the global quality of depression care [1]. Because deprexis is a fully automated digital intervention that does not burden clinicians, it can be implemented easily in different care settings. Given the robust evidence for deprexis, as shown in this updated meta-analysis, the challenge now is to implement the programme in relevant depression care settings across the globe, to continue to evaluate its effectiveness in the field, and to develop it further in order to maximize clinical benefits.

## Supporting information

S1 File (DOCX)

S1 PRISMA PRISMA 2009 checklist. (DOCX)
