# Supplementary material for: Effectiveness of a tailored, integrative Internet intervention (deprexis) for depression: Updated meta-analysis
Source: PLoS One. 2020 Jan 30;15(1):e0228100. doi: 10.1371/journal.pone.0228100 (PMC6992171; doi:10.1371/journal.pone.0228100)
Supplement: S1 File — (DOCX) [file pone.0228100.s001.docx]

**Fig 1.** Forest plot for the effectiveness of deprexis for depressive symptoms at post-intervention

**Fig 2.** Funnel plot for publication bias analysis.
